# Exploring Molecular Drivers of PARPi Resistance in BRCA1-Deficient Ovarian Cancer: The Role of LY6E and Immunomodulation

**DOI:** 10.3390/ijms251910427

**Published:** 2024-09-27

**Authors:** Tirzah Braz Petta, Joseph Carlson

**Affiliations:** 1 Department of Pathology, Keck School of Medicine, University of Southern California, Los Angeles, CA 90089, USA; 2 Centro de Biociencias, Departmento de Biologia Celular e Genetica, Universidade Federal do Rio Grande do Norte, Natal CEP 59078-970, Brazil; 3 Department of Pathology, City of Hope, Duarte, CA 91010, USA; jocarlson@coh.org

**Keywords:** ovarian neoplasms, poly(ADP-ribose) polymerase inhibitors, cellular immunity, lymphocyte antigen 6 complex, locus E, human, interferon

## Abstract

Approximately 50% of patients diagnosed with ovarian cancer harbor tumors with mutations in BRCA1, BRCA2, or other genes involved in homologous recombination repair (HR). The presence of homologous recombination deficiency (HRD) is an approved biomarker for poly-ADP-ribose polymerase inhibitors (PARPis) as a maintenance treatment following a positive response to initial platinum-based chemotherapy. Despite this treatment option, the development of resistance to PARPis is common among recurrent disease patients, leading to a poor prognosis. In this study, we conducted a comprehensive analysis using publicly available datasets to elucidate the molecular mechanisms driving PARPi resistance in BRCA1-deficient ovarian cancer. Our findings reveal a central role for the interferon (IFN) pathway in mediating resistance in the context of BRCA1 deficiency. Through integrative bioinformatics approaches, we identified LY6E, an interferon-stimulated gene, as a key mediator of PARPi resistance, with its expression linked to an immunosuppressive tumor microenvironment (TME) encouraging tumor progression and invasion. LY6E amplification correlates with poor prognosis and increased expression of immune-related gene signatures, which is predictive of immunotherapy response. Interestingly, LY6E expression upon PARPi treatment resistance was found to be dependent on BRCA1 status. Gene expression analysis in the Orien/cBioPortal database revealed an association between LY6E and genes involved in DNA repair, such as Rad21 and PUF60, emphasizing the interplay between DNA repair pathways and immune modulation. Moreover, PUF60, Rad21, and LY6E are located on chromosome 8q24, a locus often amplified and associated with the progression of ovarian cancer. Overall, our study provides novel insights into the molecular determinants of PARPi resistance and highlights LY6E as a promising prognostic biomarker in the management of HRD ovarian cancer. Future studies are needed to fully elucidate the molecular mechanisms underlying the role of LY6E in PARPi resistance.

## 1. Introduction

Ovarian cancer remains a significant challenge in oncology, with high mortality rates and limited treatment options [1]. Half of ovarian tumors are homologous recombination repair deficient (HRD) meaning they are unable to efficiently repair DNA double-strand breaks (DSBs) [2]. This molecular phenotype represents a positive predictive biomarker for the clinical use of Poly(ADP-ribose) polymerase inhibitors (PARPi) and platinum-based chemotherapy in ovarian cancers [3,4]. PARPis have transformed the ovarian cancer treatment landscape for the last decade. Patients with HRD tumors are eligible for a combination therapy of PARPi and Bevacizumab, an antiangiogenic, as maintenance treatment [5]. As for now, PARPis Olaparib, Niraparib, and Rucaparib are used as maintenance therapy in the United States. Olaparib is approved as a monotherapy for maintenance treatment in patients with a pathogenic BRCA mutation who respond well to first-line chemotherapy. In comparison, Niraparib is prescribed as maintenance therapy, regardless of HRD or BRCA mutational status, after a positive response to initial platinum-based treatment, without the addition of Bevacizumab [6,7]. However, 80% of patients with ovarian cancer suffer a recurrence within 2 years, and the overall response rate for platinum-resistant ovarian cancer to cytotoxic chemotherapy or PARPi is modest [8,9,10]. Additionally, immune checkpoint blockade (ICB) therapy as the use of anti-PDL1 or PD-1 antibodies has shown limited results in improving overall survival in patients who progressed after PARPi [11], emphasizing the importance of predictive biomarkers in clinical decision-making and further mechanistic unraveling of treatment response. Understanding the molecular mechanisms underlying PARPi resistance is essential for developing strategies to improve patient outcomes [12].

The BRCA proteins are key stabilizing factors for the maintenance of replication fork integrity following replication stress. Replication fork stabilization has recently been identified as a potential compensatory PARPi resistance mechanism [13]. There is a connection between replication stress, inflammatory signaling, and modulation of the tumor microenvironment (TME) in which the BRCA proteins seem to play different roles [14,15,16,17]. In the absence of BRCA proteins at stalled replication forks, the exonuclease MRE11 degrades single-strand DNA (ssDNA), leading to long stretches of DNA (>4–5 kb), and this is related to chemotherapeutic sensitivity regardless of the HRD status [18]. MRE11 degradation of ssDNA facilitates cGAS activation in an independent fashion of its nuclease activity, highlighting that the HR proteins are involved in more than only direct DSB repair [19].

The cGAS–STING signaling pathway has a critical role in stimulating or enhancing innate and adaptive immunity through cytokines such as type I interferon (IFN), promoting the maturation and production of immune cells such as T cells, dendritic cells, and natural killer cells to trigger effective anti-tumor immune effects [20]. BRCA1 is localized at the promoters of the molecules involved in type I IFN signaling leading to their up-regulation [21]. While the IFN pathway’s involvement in cancer immunosurveillance and response to therapy has been recognized, its specific implications in PARPi resistance, particularly in the context of BRCA1 deficiency, remain to be fully elucidated.

In the present study, we used publicly available datasets to perform a comprehensive investigation to unravel the molecular events driving resistance to PARPi therapy in BRCA1-deficient ovarian cancer. Analyses of these datasets have great potential to identify robust molecular signatures to inform clinical care and facilitate therapeutics development. Our findings shed light on the complex interplay between IFN pathway activation and DNA repair, highlighting novel candidate genes implicated in this process. We present compelling evidence implicating LY6E (lymphocyte antigen 6 complex, locus E; aliases RIGE; SCA2; RIG-E; SCA-2; TSA-1), a member of the Ly-6/uPAR protein family and is a critical mediator of PARPi resistance in BRCA1-deficient ovarian cancer. We show that LY6E expression is strongly linked to the activation of the IFN pathway, the development of resistance, and the establishment of an immunosuppressive TME. Moreover, we unveil a novel dependency of LY6E expression on BRCA1 status, further underscoring its significance in the context of PARPi response in HRD tumors. Furthermore, our analysis of Orien/cBioPortal data reveals a prognostic implication of LY6E amplification in ovarian cancer, suggesting its potential utility as a biomarker for patient stratification and therapeutic decisions. Notably, gene expression analysis implicates RAD21 and PUF60, key players in DNA repair signaling, in the regulation of LY6E expression and the development of PARPi resistance. The presence of high copy number variation (CNV) events in LY6E, RAD21, and PUF60 is related to the presence of T-cell-inflamed microenvironment, characterized by active IFN signaling, cytotoxic effector molecules, antigen presentation, and T-cell-active cytokines, a common feature of the biology of tumors that are responsive to PD-1 checkpoint blockade.

## 2. Results

### 2.1. The Interferon Alpha/Beta Signaling Pathway Plays a Central Role in PARPi Response in BRCA1-Deficient Cells

**Figure 1:**
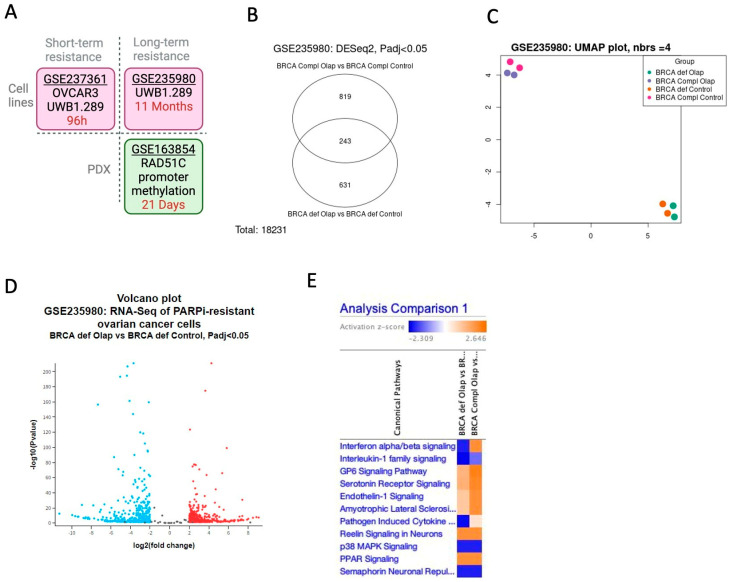
BRCA1-deficient cells resistant to Olaparib activate inflammatory response through interferon signaling pathway. (**A**) Graphic summary of the three datasets used to analyze the response to PARPi resistance in this study. 96 h is 96 hours. (**B**) Images generated using the web tool GEO2R to explore the dataset GSE235980 with a Venn diagram comparing the samples of the BRCA1-deficient (BRCA def) and BRCA1 complemented with BRCA1 gene (BRCA compl) treated with Olaparib using DESeq2 with padj < 0.05. (**C**) Uniform manifold approximation and projection for dimension reduction plot showing the distribution of the 8 samples, with the colors, as explained in this Figure legend. (**D**) Volcano plot with DEG BRCA-deficient Olaparib versus BRCA-deficient control. Red represents genes with high expression, blue represents genes with low expression, and gray represents genes with equal expression. (**E**) Canonical pathways in the comparison analysis within the 2 groups, BRCA-deficient Olaparib versus control and BRCA-complemented Olaparib versus control. Z-score cutoff: 2.

To study resistance mechanisms in ovarian cancer, we took advantage of available datasets at GEO/NCBI that performed RNAseq in HRD cells treated with PARPi in three different contexts (Table 1 and Figure 1A). To explore the molecular pathways that are differently expressed in ovarian cancer cells resistant to PARPi first, we analyzed the RNAseq results from the dataset GSE235980 that used UWB1.289 (BRCA1-deficient) and UWB1.289 + BRCA1 (BRCA1-complemented) cells treated with Olaparib for 11 months to select PARPi-resistant cells. We compared the datasets: (1) BRCA1-deficient treated with Olaparib versus BRCA1-deficient Control and (2) BRCA1-complemented Olaparib versus BRCA1-deficient cells complemented with BRCA1 gene control according to Benjamini and Hochberg (FDR), with a significance level cut-off of 0.05 and Log2FC threshold of 2. As shown in the Venn diagram, a total of 243 genes are shared between analysis 1 and 2 (Figure 1B and Supplementary Table S1). The PCA analysis revealed that the groups BRCA1-deficient and -complemented segregated independently (Figure 1C). The volcano plot highlights the distribution of the fold changes in each differentially expressed gene in BRCA1-deficient treated with Olaparib (Figure 1D). This gene set was further used for functional enrichment analysis using the comparison analysis in ingenuity pathway analysis (IPA, Qiagen). We first manually filtered genes involved in DNA repair canonical pathways, and we could not find any differences between the BRCA1-deficient and BRCA1 complemented resistant to Olaparib after 11 months groups. In the Canonical Pathway analysis (only *p*-value log10 cutoff 1.3 and z-score absolute value cutoff 2), the heatmap shows that the pathway with the highest *p*-value differences between Olaparib treatment of BRCA1-deficient and -complemented cells is Interferon alpha/beta signaling, with inhibition of the genes in BRCA1-deficient cells (Figure 1E). It is worth citing that the paper describing the results from the dataset GSE237361 found the same activation of ISGs and cGAS–STING pathway after Olaparib treatment [22].

### 2.2. BRCA1-Deficient Ovarian Cancer Cells Resistant to Olaparib Present a Positive Regulation of Cytokine as Interferon Production with Overexpression of LY6E

**Figure 2:**
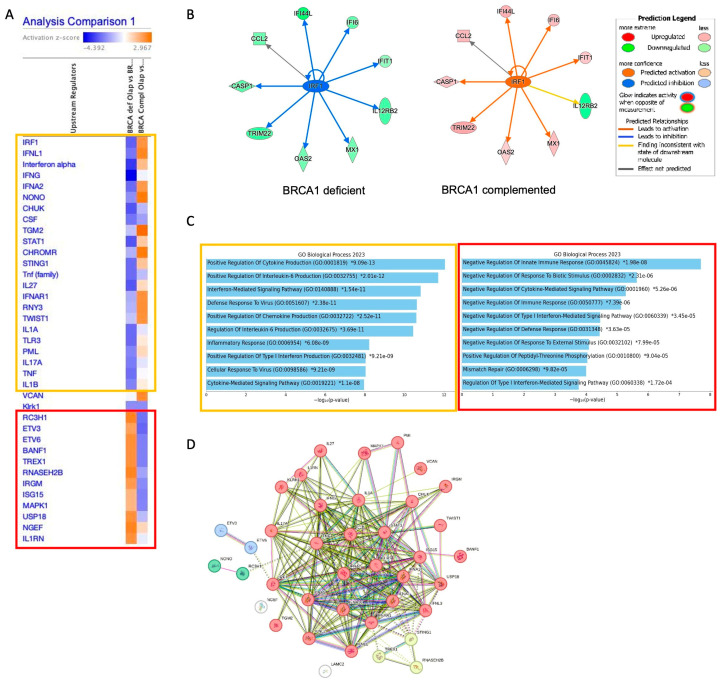
IPA in PARPi-resistant cells. (**A**) Upstream regulators in the comparison analysis within the 2 groups, BRCA-deficient Olaparib versus control and BRCA-complemented Olaparib versus control. Z-score cutoff: 2. (**B**) Network generated from Figure 1A where we can see all the genes present in the dataset that are related to IRF1 and their impact (activation or inhibition) as displayed in the legend. (**C**) GO analysis with the pathways with positive or negative regulation in the two groups from Figure 1A. The orange rectangle with genes activated in the group BRCA-complemented and the red rectangle in the group BRCA-deficient. (**D**) PPI network using the list of genes upstream regulators generated using STRING database. The core with the highest FDR (FDR = 4.87 × 10^−13^) according to KEGG is related to Cytosolic DNA-sensing pathway (hsa04623). MCL clustering analysis highlights the genes STING1, TREX1, and RNASEH2B, in yellow, as part of this network.

We explored the dataset using IPA to find the most significant differences in the upstream regulators, and we observed the transcription factor IRF1 as the most statistically differently expressed upstream regulator among the 2 groups (Figure 2A). In the BRCA1-deficient group, IRF1 is predicted to be inhibited, and this leads to less activation of its targets, such as the peptidase CASP1, the IFN-stimulated genes (ISGs) MX1 and OAS2, the cytokine CCL2, the transmembrane receptor IL12RB2, and the interferon-stimulated genes: IFI6, IFI44L, and IFIT1 (Figure 2B). In the presence of BRCA1, we observed the opposite tendency, with activation of the genes in this network. The IL12RB2 gene did not alter its expression according to BRCA1 status, and according to the IPA database, this finding is inconsistent with the state of a downstream molecule, showing the importance of this protein in the response to PARPi. IL12RB2 is a transmembrane receptor that promotes the proliferation of T-cells and NK cells and induces the promotion of T-cells by strongly enhancing IFN-gamma production, an antagonist to IFN-alpha and beta.

Next, we performed GO analysis of the genes activated or inhibited in the upstream regulator analysis by dividing them into 2 groups: (i) highlighted in the orange rectangle, genes activated in the BRCA1-complemented analysis and inhibited in BRCA1-deficient, and (ii) in the red rectangle, genes inhibited in BRCA1 complemented analysis and activated in BRCA1-deficient. In the Figure 2C we observe that in the GO analysis in the orange rectangle there is an enrichment for positive regulation of cytokine and interleukin production, whereas in the second GO analysis, there is a negative regulation of these pathways. This analysis suggests that BRCA1-deficient cells resistant to Olaparib present an immunosuppressive response.

Interestingly, when we analyzed the PPI relationship among all upstream regulator genes, we observed a network with 4 main clusters, with INF-mediated pathways being the most representative (Figure 2D, red cluster) and Cytosolic DNA-sensing pathway (hsa04623, in yellow cluster) being the second, with a high FDR (FDR = 4.84 × 10^−13^).

In the yellow cluster, we observed the gene RNASEH2B. A recent study used CRISPR-Cas9 screens to identify a PARPi sensitivity marker and found that alterations in the genes encoding the RNase H2 enzyme complex (RNASEH2A, RNASEH2B, and RNASEH2C) cause PARPi sensitivity of cells via impaired ribonucleotide excision repair [23]. We observed that the expression of RNASEH2A is increased in BRCA1-deficient cells, but its expression does not change following Olaparib treatment (Supplementary Figure S1). Conversely, we observed an inverted change in the expression of the gene RNASEH2C in BRCA-deficient cells compared to the complemented (Supplementary Figure S1). 

Altogether these results suggest that BRCA1-deficient ovarian cancer cells resistant to Olaparib trigger an overall negative regulation of the immune response that has a relationship with the presence of cytosolic cytoplasmic ssDNA and activation of the STINGs (Stimulator of Interferon Genes) pathway.

### 2.3. STING Response to Olaparib Requires BRCA1 but cGAS Downregulation Does Not

Dysregulation of the cGAS–STING pathway has been implicated in ovarian cancer, where alterations in DNA repair mechanisms and genomic instability can lead to the accumulation of ssDNA, activating the cGAS–STING pathway [24,25]. cGAS synthesizes cyclic GMP–AMP (cGAMP), which serves as a second messenger that relays its signal to downstream innate immune responses through STING [24]. The induction of the tumor-intrinsic STING pathway activity subsequently stimulates secretion of IFNs from cancer cells. Activation of this pathway can influence the TME and impact tumor progression and response to therapy.

**Figure 3:**
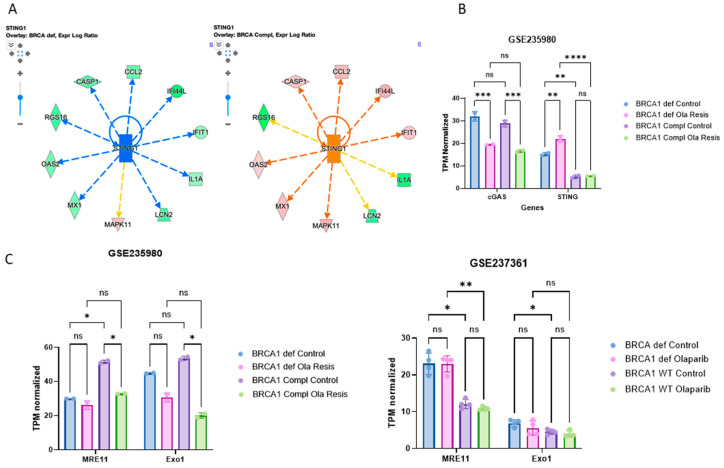
The role of DNA exonucleases and cGAS–STING pathway in PARPi resistance. (**A**) Network with STING1 expression in BRCA1-deficient and BRCA1-complemented cells, with their relationship with other molecules as explained in the predict legend on the upper right corner. Dashed lines predict indirect relationships. (**B**) Gene expression level as TPM normalized in the dataset GSE235980 of the genes cGAs and STING. See the legend in this Figure for the colors of the bars. Two-way ANOVA multiple comparison test with adjusted *p*-value applied to the asterisks. (**C**) Gene expression level as TPM normalized in the dataset GSE235980 and GSE237361 of the genes MRE11 and Exo1. See the legend in this Figure for the colors of the bars. Two-way ANOVA multiple comparison test with adjusted *p*-value applied to the asterisks. Ns = *p* > 0.05; * *p* ≤ 0.05; ** *p* ≤ 0.01; *** *p* ≤ 0.001 and **** *p* ≤ 0.0001.

In our analysis, we observed that in BRCA1-complemented cells resistant to Olaparib, STING1 is downregulated, and the opposite is observed in BRCA-deficient cells (Figure 3B). The downstream regulators impacted by STING1 expression level are closely related to the genes regulated by IRF1 in Figure 2B. This finding follows the literature since one of the major consequences triggered by the STING pathway is the production of cytokines of the type I interferon family, which in turn induces the expression of hundreds of interferon-stimulated genes (ISGs), among those, LY6E [26,27,28].

In the BRCA1-deficient cells treated with Olaparib, cGAS is less expressed than the control, whereas STING is overexpressed (Figure 3B) following treatment. cGAS downregulation in Olaparib-resistant cells seems to be independent of BRCA1; however, STING1 expression is highly decreased in the presence of BRCA1 (Figure 3B). Even though we cannot assume their expression is related to their activation, cGAS-independent STING activation has been shown in response to DNA damage [29,30].

There is emerging evidence suggesting an interplay between the cGAS–STING pathway and the HR protein MRE11 in tumorigenesis [31]. MRE11 is a component of the MRN complex (MRE11–RAD50–NBS1), which plays a crucial role in DNA damage signaling, HR, and maintenance of genome stability [31]. In the absence of BRCA1, stalled replication forks are extensively degraded by the nucleases MRE11 together with EXO1, leading to chemotherapeutic sensitivity [32]. In Olaparib-resistant BRCA1-complemented cells, MRE11 is overexpressed compared to BRCA-deficient. Both exonucleases MRE11 and EXO1 are less expressed in the presence of BRCA1 in Olaparib-resistant cells (Figure 3C, left panel). However, in BRCA-deficient cells treated with Olaparib for a shorter period, MRE11 expression increases, whereas EXO1 is not altered (Figure 3C, right panel. This difference in their expression suggests an adaptative response that is related to the resistance mechanism over time as suggested previously [33,34].

In long-time Olaparib resistance, both MRE11 and EXO1 are downregulated in BRCA1-deficient cells. It suggests that these cells might have evolved mechanisms to circumvent the resolution of stalled replication forks. In short-term treatment, MRE11 is upregulated in BRCA-deficient cells, suggesting a potential increase in activity of this nuclease at stalled replication forks.

### 2.4. LY6E Amplification Is Also Present in PDX HRD PARPi-Resistant Tumors

**Figure 4:**
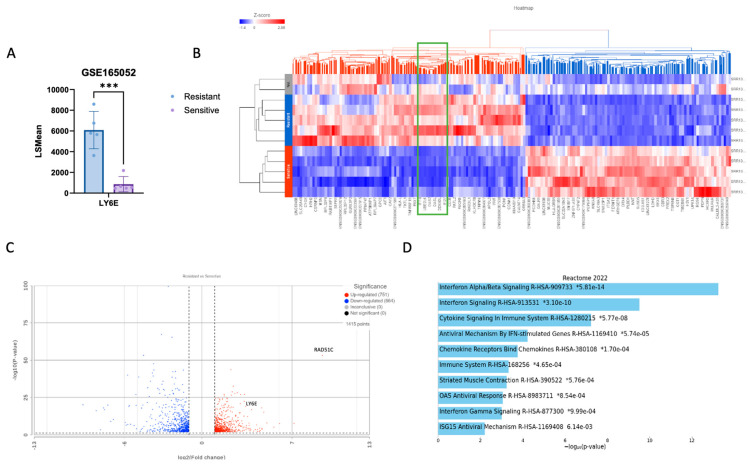
HRD Niraparib-resistant vs. -sensitive PDX tumors RAD51-deficient (from the dataset GSE165054). (**A**) Expression level of the gene LY6E in resistant and sensitive phenotype. *** *p* ≤ 0.001. (**B**) Heatmap showing two clusters (red and blue) according to the z-score of the phenotypes Sensitive or Resistant. The green rectangle highlights the genes IFI27, OAS2, and IFI16. (**C**) Volcano plot with the 1415 genes with FC < −2 and >2, *p*-value < 0.05, highlighting the LY6E and RAD51C genes. (**D**) GSEA analysis of top 100 DEGs comparing Sensitive x Resistant. Reactome, EnrichR.

We explored the RNAseq from another dataset containing PARPi-sensitive PDX tumors with RAD51C promoter methylation and further PARPi resistance with the reversion of RAD51C promoter methylation and reactivation of the gene expression (GSE165054). We observed a 7-fold difference in the LY6E gene expression when comparing Niraparib-resistant (LSMean 6085.03) to -sensitive (LSMean 866.46) (*p*-value 1.09 × 10^−6^) (Figure 4A). The ISGs IFI27 (interferon-inducible protein 27) and IFI16 (interferon-inducible protein 16) are also upregulated in the resistant group by 5-fold (*p*-value 9.51 × 10^−4^) and 7-fold (*p*-value 2.3 × 10^−12^), respectively. The OAS2 gene found in networks in Figure 2B and Figure 3A is also highly expressed in resistant tumors (see green rectangle in Figure 4B). It has been shown that STING can be activated in a non-canonical manner by IFI16 together with the DNA repair proteins ATM (ataxia telangiectasia mutated) and PARP1 [35]. IFI16 shuttles between the nucleus and the cytosol and also helps cGAS in the activation of STING during cytosolic DNA recognition in human cells. However, even though IFI16 is also involved in conventional cytosolic DNA sensing, it promotes a different mode of STING activation after DNA damage-inducing. Reinforcing the hypothesis of an alternative STING pathway upon Olaparib resistance. Also, it has been suggested by others that STING agonism reprograms myeloid cells in the TME of PARPi-resistant ovarian tumors and overcomes the resistance [36].

We analyzed the differently expressed genes in resistant vs. sensitive (2 < FC > 2 and *p*-value less than 0.05), and we found 751 up-regulated genes and 664 downregulated (Figure 4C). RAD51C was the gene with the highest-fold change (649.69) due to acquired PARPi resistance and loss of RAD51C methylation. Gene enrichment analysis of the 100 genes with the highest fold change ratio and *p*-value shows activation of pathways related to interferon Alpha/Betta signaling (Figure 4D).

### 2.5. LY6E Is Amplified in Ovarian Cancer and Acts a Prognostic Biomarker

**Figure 5:**
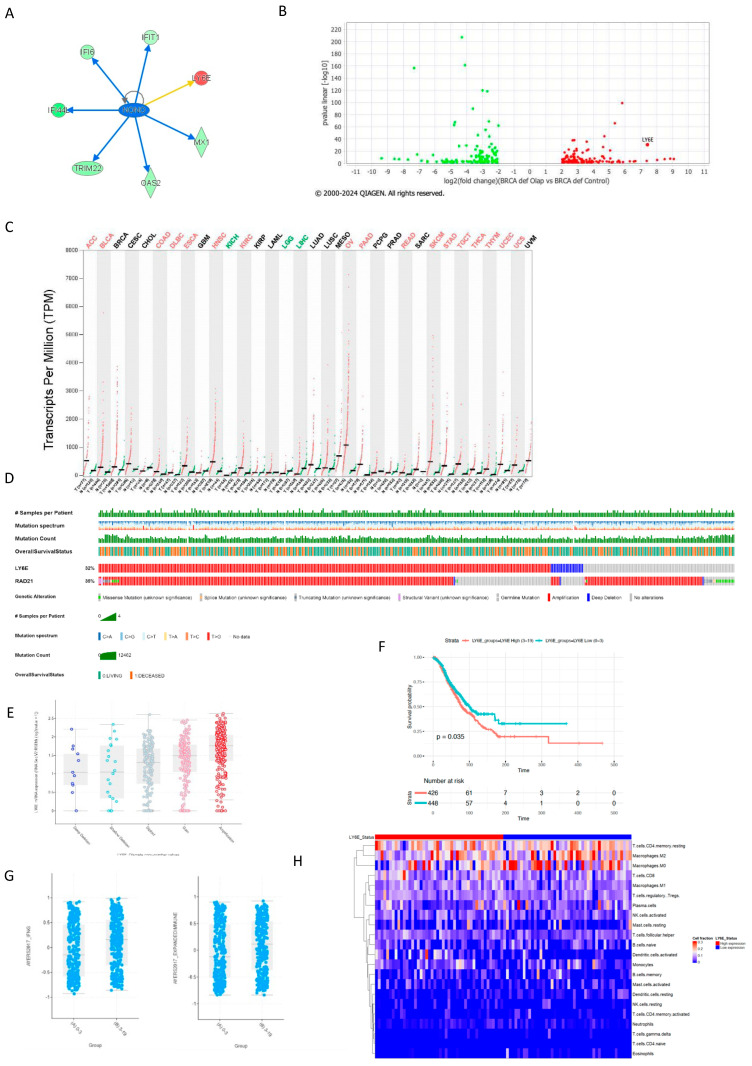
The LY6E as a prognostic biomarker for ovarian cancer. (**A**) PPI network. (**B**) Volcano plot from BRCA1-deficient cells resistant to Olaparib from the dataset GSE235980 using the 243 DEG used in the first analysis. The gene LY6E is highlighted. (**C**) Tissue-wise expression profile of LY6E in different cancer types using a dot plot according to data in GEPIA2 for normal tissues (green) and tumor (red). The highest expression is observed in ovarian cancer. Abbreviated cancer names are as per TCGA. (**D**) Oncoplot of ovarian tumors for LY6E and RAD21. The legend is displayed in this Figure. (**E**) LY6E mRNA expression in ovarian cancer according to the type of genetic mutation (deep deletion, shallow deletion, diploid, gain, and amplification. Each point represents one tumor. The legend is displayed in this Figure. (**F**) Kaplan–Meier curve with overall survival of patients with ovarian cancer distributed according to CNV in the gene LY6E as low ((**A**) 0 to 3 events) or high ((**B**) 3 to 19 events). *p*-value is displayed in this Figure’s panel. (**G**) AYERS2017_IFNG signature of ovarian tumors classified according to the number of CNV events in LY6E as (**A**) low or (**B**) high. *p*-value = 4.048 × 10^−4^, Wilcoxon test. AYERS2017_EXPANDEDIMMUNE, *p*-value 7.695 × 10^−3^. (**H**) CIBERSTORTx analysis of TCGA Ovarian Tumors according to the expression of the gene LY6E divided into Low and High according to the median of the z-score.

Among the top upstream regulators in BRCA1-deficient cells resistant to Olaparib, we observe the inhibition of the transcription factor NONO among the ISGs (Figure 2A). This observation caught our attention since this gene is involved in DNA non-homologous end joining (NHEJ) required for double-strand break repair and V(D)J recombination and may stabilize paired DNA ends and also in the regulation of cGAS–STING pathway [37,38]. The NONO network in BRCA1-deficient cells predicts inhibition of ISGs and activation of LY6E, which is a finding inconsistent with IPA’s database (Figure 5A), suggesting a specific expression of LY6E in the context of PARPi resistance. Interestingly, the gene LY6E is one of the top 5 up-expressed genes in BRCA-deficient cells resistant to Olaparib (Figure 5B).

Our analysis shows that across all tumor types, ovarian cancer presents the highest LY6E expression (GEPIA2) (Figure 5C). The LY6E gene is mutated in 32% of ovarian tumors in the cBioPortal (total 1018 patients and 2141 samples) (Figure 5D) and amplification is the most common mutation, which is reflected in increasing mRNA expression (Figure 5E). Additionally, ovarian cancer patients with high CNV copies and high gene expression of LY6E present a worse OS (Log rank *p*-value 0.02) (Figure 5F). A previous study [31] found that patients with high LY6E expression had poorer overall survival (OS). This overexpression of LY6E may suppress T cell responses, contributing to immune suppression and potentially leading to worse clinical outcomes.

Considering IFNG as a critical driver of programmed death ligand-1 (PD-L1) expression in cancer and host cells, and baseline intra-tumoral T cell infiltration may improve response likelihood to anti-PD-1 therapies, we investigated the expression of the predict signatures developed by Ayers et al. [39]. Interestingly, patients with high CNV presented a higher IFNG and expanded immune signature, and these signatures are related to good response to ICB (Figure 5G). It has been described that LY6E promotes cytokine-induced PD-L1 expression and activation and binding of NK cells to cancer cells [40]. Most of the patients from ORIEN/cBioPortal database did not receive ICB therapy, thus this dataset is not suitable to test LY6E as a prognostic biomarker. Interestingly, according to the Human Protein Atlas single-cell database, immune cells in the ovary highly express Ly6E (Supplementary Figure S2).

We further inferred the composition of 22 immune cell subsets in the TCGA_OvarianCancer after dividing the samples into low and high LY6E expression. The results were generated using CIBERSORTx, and the built-in LM22 immune cell gene signature shows that the group high are less enriched for cell fractions containing T cells CD4+ memory resting, Macrophages M2 (anti-inflammatory), and Macrophages M0 (non-activated). Depending on the activation pathways, M0 can differentiate into two activated subtypes, M1 and M2, which each exhibit distinct immunoregulatory roles. CD4+ T cells are central players and coordinators of innate and antigen-specific immune responses [40,41]. Memory CD4+ T cells initiate a reaction to reinfections that is quicker and of a higher magnitude than primary responses and can contribute to protective immunity. CD4+ memory T cells are an antigen-experienced population that can have a role in neoantigen expression in tumor cells [42]. The expression of LY6E did not impact OS in the same dataset of patients (Figure 1B). Previous work has shown that niraparib treatment increases the activity of type I (alpha) and type II (gamma) interferon pathways and enhances the infiltration of CD8+ cells and CD4+ cells in tumors [43].

Interestingly, a recent work shows that neutrophils with high expression in Ly6E not only act as a biomarker but also function as an immunomodulator sensitizing melanoma-resistant tumors to anti-PD1 therapy, in part, by creating an environment permissive to CD8+ T cell activation through secretion of known activating factors such as IL-12b [44,45].

### 2.6. LY6E and Rad21 Share Correlation Expression in Ovarian Tumors

**Figure 6:**
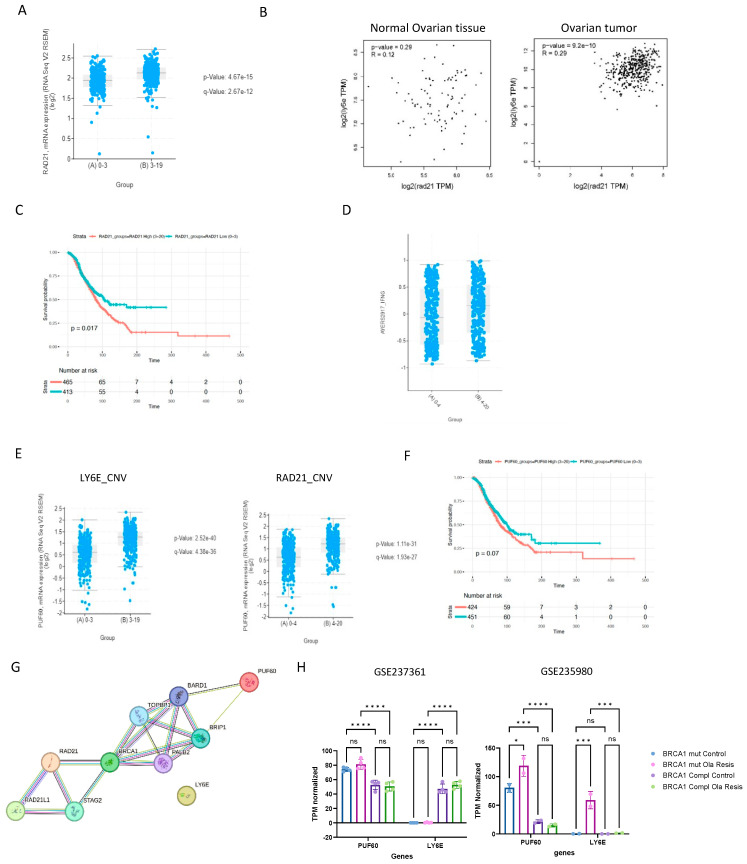
The co-expression of LY6E, PUF60, and RAD21 are important for PARPi resistance. (**A**) RAD21 mRNA expression according to LY6E CNV groups low or high. *p*-value are indicated in this Figure. (**B**) Pearson correlation coefficient of the genes LY6E and RAD21 in normal tissue (R = 0.12; *p*-value = 0.29) and ovarian tumors (R = 0.29; *p*-value = 9.2 × 10^−10^). (**C**) Kaplan–Meier curve with overall survival of ovarian cancer patients according to RAD21 CNV low (0 to 3 events) or high (3 to 20 events). Log rank *p*-value is displayed in this Figure. (**D**) AYERS2017_IFNG signature in ovarian tumors divided in low and high RAD21 CNV. *p*-value = 1.145 × 10^−3^. (**E**) mRNA of the gene PUF60 in ovarian tumors according to low or high CNV in the genes LY6E and RAD21. *p*-values are indicated in this Figure. (**F**) Kaplan–Meier curve with overall survival of patients according to PUF60 CNV low (0 to 3 events) or high (3 to 20 events). (**G**) PPI network analysis in STRING formed by the proteins LY6E, RAD21, PUF60. Nodes and edges colors are as per STRING legend pattern. (**H**) Gene expression level as TPM normalized in the dataset GSE237361 and GSE235980 of the genes PUF60 and LY6E. See the legend in this Figure for the colors of the bars. ns = *p* > 0.05; * *p* ≤ 0.05; *** *p* ≤ 0.001 and **** *p* ≤ 0.0001.

Next, we explored the pattern of overall gene expression in both groups with LY6E low-CNV and high-CNV using data from ORIEN/cBioPortal. The gene with the highest expression in the group LY6E high-CNV was Rad21 (sister chromatid cohesion protein 1) (Figure 6A). Interestingly, this expression association is not seen in normal ovarian tissue (GEPIA2) (Figure 6B).

We then inquired about the rate of Rad21 mutation in ovarian tumors, and as you can see in the oncoplot from Figure 5D, Rad21 is mutated in 35% of all ovarian tumors, with mostly gene amplification events. When analyzing ovarian tumors according to the number of Rad21 CNV (0–4 low and 4–20 high), we observed that the number of Rad21 copies is related to a worse prognosis in this type of cancer (Figure 6C). High CNV is also related to a high IFN-G immune signature, suggesting these patients are probably good responders for PD-L1 blockade therapy (Figure 6D). We analyzed the gene expression in the group of Rad21 high-CNV (group (B) 4–20), and the gene with the highest co-expression *p*-value was PUF60, which also presented a higher correlation with high LY6E CNV (group (B) 3–19) (Figure 6E). PUF60 (Poly(U) Binding Splicing Factor 60) DNA- and RNA-binding proteins have been implicated in cellular processes such as DNA repair, transcriptional regulation, and mRNA stability. Interestingly, CNV and/or elevated expressions of PUF60 have been reported in multiple cancers, and thus cancer-promoting functions have been proposed for PUF60 [46]. Its high expression is closely associated with the high incidence of lymph node metastasis and advanced TNM stage in breast cancer [47]. Here, we show that PUF60 high CNV is associated with a tendency for worse survival outcomes in ovarian cancer (Figure 6F). We investigated the expression of these genes in a subset of ovarian cancer cell lines, and the same pattern was observed, where RAD21 CNV is strongly associated with PUF60 and LY6E (Pearson 0.76, Spearman 0.79, *p*-value 1.56 × 10^−140^ and Pearson 0.74, Spearman 0.76, *p*-value 7.80 × 10^−14^, respectively) (Supplementary Figure S2). The genes Rad21 and PUF60 are connected via BRCA1 in the biological process of establishment of meiotic sister chromatid cohesions (GO:0034089) and replication-related DSB repair (GO:1990414) (Figure 6G). However, LY6E is not directly related to this network (text mining was removed to build this network), suggesting that the connections between these genes are not direct and should be further investigated.

PUF60, Rad21, and LY6E are genes located on chromosome 8q24, an important locus associated with the progression of ovarian cancer and amplified region in around 14% of ovarian tumors [47,48]. Amplification of the 8q24 locus was also observed in prostate, colorectal, breast, bladder, and lung cancers. Other genes from the LY6 family are also located at the 8q24 loci, LY6H, LY6K, LY6D, LY6L. However, these genes are not explored here, as their expression did not change under PARPi sensitivity status according to our analysis (see Supplementary Table S1). 

Using the gene expression information from the dataset GSE235980, we observed that the expression of PUF60 is increased in Olaparib-resistant BRCA1-deficient cells, and this was not observed in cells treated with Olaparib for a short period (GSE237361). Surprisingly, the expression of LY6E is dependent on BRCA1 in the dataset GSE237361, and cells resistant to Olaparib re-express LY6E in the dataset GSE235980. This finding emphasizes once again the importance of LY6E in PARPi resistance in the absence of BRCA1, and this mechanism should be better characterized (Figure 6H).

## 3. Discussion

Here, we present a comprehensive analysis exploring the mechanisms underlying PARPi resistance in HRD ovarian cancer in a cellular model and PDX. We identified a dynamic interplay between DNA repair pathways and immune response modulation. Our findings highlight the central role of the interferon alpha/beta signaling pathway in mediating PARPi resistance in BRCA1-deficient cells. Notably, resistance to Olaparib is associated with dysregulated cytokine production, particularly interferon target genes, evidenced by the overexpression of LY6E. LY6E overexpression has been associated with drug resistance in several cancers, such as breast cancer, gastric cancer, and lung cancer [49]. 

LY6E interacts with a range of immune cells, including T cells, B cells, dendritic cells, and tumor cells, and these interactions can influence immune checkpoint signaling and contribute to immune suppression [50]. LY6E and IFI27 are also upregulated during immune activation, particularly through pathways like cGAS–STING, which promote interferon production [51]. Additionally, IFI27 is predominantly localized to the mitochondrial membrane [52]. It plays a role in mitochondrial-mediated apoptosis by disrupting mitochondrial integrity, leading to cell death in response to infection or stress [53]. In PARPi-resistant ovarian cancer cells, altered mitochondrial dynamics and energy metabolism often occur as adaptive mechanisms to maintain survival [54]. The overexpression of IFI27 has been linked to drug resistance in cancer and resistance to apoptosis, which allows cancer cells to survive despite chemotherapy [54]. This resistance can occur because: (i) IFI27 overexpression may affect mitochondrial integrity differently in cancer cells, making them less prone to apoptotic signaling; (ii) some studies suggest that IFI27 may inhibit the apoptotic pathway in certain cancer cells, promoting survival even in the presence of DNA-damaging agents. Moreover, LY6E can affect the mitochondrial-mediated apoptotic pathways, making cancer cells more resilient to treatment. 

IFI27 is involved in the nuclear DNA damage response and can interact with RAD21 in the context of DNA structure and repair [55]. Rad21 is a structural component of the cohesin complex. Rad21 is important to allow correct chromosome segregation and post-replicative DNA repair by preventing inappropriate recombination between repetitive regions [56,57]. A potential role of Rad21 as a predictive and prognostic marker in BRCA-mutated patients has been suggested [58].

Additionally, the gene expression of LY6E is related to the expression of RAD21 and PUF60 [59,60], both genes are located at chromosome 8q24, a loci frequently amplified in ovarian cancer. Together, these proteins contribute to the complex interplay between genome integrity and immune sensing in the context of DNA damage.

Here we show STING response to Olaparib appears to be dependent on BRCA1 status, while cGAS downregulation is observed independently of BRCA1. cGAS and STING can function independently within the DNA sensing pathway, despite their typical collaboration in immune responses to cytosolic DNA [25,29,30]. cGAS is primarily responsible for detecting cytosolic dsDNA and synthesizing the second messenger cGAMP, which activates STING. However, cGAS-independent activation of STING has been observed under certain conditions, such as in response to other cyclic dinucleotides or damaged mitochondria [56]. Similarly, cGAS downregulation can occur without affecting STING activity, indicating that other factors or signals can stimulate STING independently of cGAS. These uncoupling roles suggest that STING can integrate diverse signals beyond cGAS-derived cGAMP, while cGAS may modulate immune responses through additional pathways or regulatory mechanisms independent of STING activation.

Finally, LY6E emerges as a promising prognostic biomarker, with its amplification correlating with worse overall survival in ovarian cancer patients and its association with immune signatures suggesting a potential role in predicting response to ICB therapy. Further investigations into the functional implications of LY6E as a prognostic biomarker for HRD-resistant ovarian tumors are warranted to translate them into clinical applications and improve patient outcomes.

## 4. Material and Methods

### 4.1. ORIEN/cBioPortal

We accessed cBioPortal through ORIEN (Oncology Research Information Exchange Network) (https://www.cbioportal.org/). The selecting Data Parameter was Gyn Cancer/Ovarian Cancer; we inquired the molecular profiles (e.g., mutations, copy number alterations, gene expression), and clinical data (e.g., patient demographics, survival outcomes). A total of 1026 patients and 2158 samples were analyzed. We used the interactive visualization tools within cBioPortal to explore the selected data parameters; to generate plots, such as mutation frequency, copy number alteration plots, and survival curves; to visualize the genomic landscape of the selected cancer cohort. After this, we performed a clinical correlation analysis between genomic alterations and patient survival. All data access, sharing, and use are consistent with the applicable Total Cancer Care Protocol and the applicable patient informed consent.

Somatic Copy Number Variation (CNV) was calculated by Sequenza (v 3.0.0, https://sequenzatools.bitbucket.io/#/home). The Sequenza seqz_ps.gz file is generated on the alignments of tumor and germline by sequenza-utils. The seq_ps.gz is processed by R script with libraries (Sequenza v3.0.0, CNTools v1.30.0), T<tumorID>_N<germlineID> after filtering false 0 CNV value, and graphic outputs. Data sharing complies with the ORIEN agreement. Any requests for raw data should be directed to ORIEN by the investigator.

### 4.2. Gene Expression Omnibus (GEO) Database (NCBI)

The NIH GEO (http://www.ncbi.nlm.nih.gov/geo/) hosted by the National Center for Biotechnology Information (NCBI), stands as one of the largest repositories of publicly available genomic data. We performed secondary analysis of existing data to explore PARPi resistance mechanisms in ovarian cancer. The dataset selection was made using the search for the words “Parp inhibitor” and “BRCA1” and “Ovarian Cancer” in the GEO database. We ensured that it includes human gene expression data by RNA sequencing data and associated metadata (e.g., sample characteristics, experimental conditions). We found and downloaded 3 profile datasets with GEO accession numbers: GSE237361, GSE235980, GSE163854 (see Table 1). We performed data preprocessing steps, including quality control, normalization, and batch effect correction, to ensure the integrity and comparability of gene expression profiles across samples. We used the interactive web tool GEO2R to analyze the groups in each dataset to identify genes expression and download tables with genes to be analyzed in IPA. In GEO2R, we used Log2FC threshold 2 and significance level cut-off 0.05 in *Options*. We generated summary statistics, visualizations (e.g., histograms, box plots), and clustering analyses to assess the distribution of gene expression values and identify potential outliers or patterns within the data. We downloaded tables from comparisons between PARPi response according to the Venn diagram in Figure 1 and used the genes shared to be analyzed in IPA. The data format from GEO dataset had to be adjusted to serve as input to IPA Core Analysis. Specifically, the *p*-value was in −log10(*p*-value), and we converted it to linear *p*-value using this Equation: [=10^(value)]. For functional gene enrichment analysis and Gene Ontology to elucidate biological processes, pathways, or molecular functions associated with DEG identified we used EnrichR website https://maayanlab.cloud/Enrichr/ (accessed on 22 January 2024).

**Table 1:** Description of the datasets containing experiments with ovarian cancer, HRD and Parp inhibitor.

GEO NCBI	PARPi	Treatment Period	Cells	Reference
GSE237361	Olaparib	96 h (short-time resistance)	UWB1.289 (BRCA-deficient) Ovca3 (BRCA WT)	[18]
GSE235980	Olaparib, Niraparib, Talazoparib, Rucaparib, Veliparib	11 months (long-time resistance)	UWB1.289 (BRCA-deficient) UWB1.289 + BRCA1 (BRCA-complemented)	[61]
GSE165054	Niraparib	21 days (long-time resistance)	PDX Rad51C promoter methylation (Resistant X Sensitive)	[62]

### 4.3. Gene Expression Analysis

To assess the expression of the gene of interest, we used the “Profile graph” function in the web tool GEO2R using the GEO accession number and defining the groups according to the metadata (BRCA1-deficient, BRCA1 WT, BRCA1-complemented). The TPM-normalized expression value of the gene-across samples was analyzed using GraphPad Prism version 10.0.0 for Windows, GraphPad Software, Boston, MA, USA, www.graphpad.com.

### 4.4. Ingenuity Pathway Analysis (IPA) QIAGEN

Datasets were analyzed with the use of IPA. The networks and functional analyses were generated through the use of IPA (QIAGEN Inc., Hilden, Germany (https://digitalinsights.qiagen.com/IPA) (accessed on 12 March 2024). The IPA predicted which upstream regulators are activated or inhibited to explain the up-regulated and down-regulated genes observed in the studied datasets. Knowledge of this regulatory cascade helped to understand the biological activities occurring in ovarian tumors or cells [63]. The cut-off used for IPA was *p* < 0.05. Gene expression data are presented as fold change (FC) on a log2 scale, where log2FC = 1 and −1 define a 2-fold increase and decrease, respectively. Activation z-scores are calculated via the IPA software and predicted whether a specific pathway, disease, or function is increased (z-score > 2.0) or decreased (z-score < −2.0) based on the experimental dataset.

### 4.5. GEPIA2

GEPIA2 (Gene Expression Profiling Interactive Analysis) (http://gepia.cancer-pku.cn/) webserver was used for gene expression analysis based on ovarian tumor and normal samples from the Cancer Genome Atlas (TCGA) and the Genotype-Tissue Expression (GTEx) [64].

### 4.6. Partek Flow v.11.0.24.0102

Partek Flow was used to analyze the dataset PRJNA693039 from GSE165052. We compared five samples with RNAseq from Niraparib-sensitive versus five samples of Niraparib-resistant. Two samples with mixed phenotypes were excluded from the analysis.

### 4.7. Protein–Protein Interaction Network Analysis

The STRING database (http://string-db.org) was used for protein–protein association networks and functional enrichment analyses of proteins of interest [65]. A degree > 10 was set as the cut-off threshold. The top 20 genes were selected as hub genes.

### 4.8. Ciberstortx

CibersortX was used to explore cell fractions on immune cell subpopulations, and we classified the tumor types as the following: (i) hot tumors: T cells CD8+ ≥ 5%; (ii) cold tumors: T cells CD8+ < 5% [66].

## Data Availability

The data from human cell lines treated with Olaparib used in this study is already available (see Material and Methods). The patient data from cBioPortal requests for raw data should be directed to ORIEN by the investigator.

## Supplementary Materials

The following supporting information can be downloaded at: https://www.mdpi.com/article/10.3390/ijms251910427/s1.

## References

[1] Jayson G.C., Kohn E.C., Kitchener H.C., Ledermann J.A. (2014). Ovarian Cancer. Lancet.

[2] Domchek S.M., Aghajanian C., Shapira-Frommer R., Rini B.I., Goss G., Hensley M.L., Schmutzler R.K., Gelmon K., Robson M.E., Olopade O.I. (2016). Inhibition of Poly(ADP-Ribose) Polymerase in Patients with Breast and Ovarian Cancer. N. Engl. J. Med..

[3] Jiang X., Li W., Li X., Bai H., Zhang Z. (2019). Current Status and Future Prospects of PARP Inhibitor Clinical Trials in Ovarian Cancer. Cancer Manag. Res..

[4] Mirza M.R., Pignata S., Ledermann J.A. (2018). Latest Clinical Evidence and Further Development of PARP Inhibitors in Ovarian Cancer. Ann. Oncol..

[5] Rose M., Burgess J.T., O’Byrne K., Richard D.J., Bolderson E. (2020). PARP Inhibitors: Clinical Relevance, Mechanisms of Action and Tumor Resistance. Front. Cell Dev. Biol..

[6] Ray-Coquard I., Pautier P., Pignata S., Pérol D., González-Martín A., Berger R., Fujiwara K., Vergote I., Colombo N., Mäenpää J. (2019). Olaparib plus Bevacizumab as First-Line Maintenance in Ovarian Cancer. N. Engl. J. Med..

[7] Moore K., Colombo N., Scambia G., Kim B.-G., Oaknin A., Friedlander M., Lisyanskaya A., Floquet A., Leary A., Sonke G.S. (2018). Maintenance Olaparib in Patients with Newly Diagnosed Advanced Ovarian Cancer. N. Engl. J. Med..

[8] Moore K.N., Secord A.A., Geller M.A., Miller D.S., Cloven N., Fleming G.F., Wahner Hendrickson A.E., Azodi M., DiSilvestro P., Oza A.M. (2019). Niraparib Monotherapy for Late-Line Treatment of Ovarian Cancer (QUADRA): A Multicentre, Open-Label, Single-Arm, Phase 2 Trial. Lancet Oncol..

[9] Gonzalez-Angulo A.M., Timms K.M., Liu S., Chen H., Litton J.K., Potter J., Lanchbury J.S., Stemke-Hale K., Hennessy B.T., Arun B.K. (2011). Incidence and Outcome of BRCA Mutations in Unselected Patients with Triple Receptor-Negative Breast Cancer. Clin. Cancer Res..

[10] Ledermann J., Harter P., Gourley C., Friedlander M., Vergote I., Rustin G., Scott C.L., Meier W., Shapira-Frommer R., Safra T. (2014). Olaparib Maintenance Therapy in Patients with Platinum-Sensitive Relapsed Serous Ovarian Cancer: A Preplanned Retrospective Analysis of Outcomes by BRCA Status in a Randomised Phase 2 Trial. Lancet Oncol..

[11] Matulonis U.A., Shapira R., Santin A., Lisyanskaya A.S., Pignata S., Vergote I., Raspagliesi F., Sonke G.S., Birrer M., Sehouli J. (2020). Final Results from the KEYNOTE-100 Trial of Pembrolizumab in Patients with Advanced Recurrent Ovarian Cancer. J. Clin. Oncol..

[12] Jackson L.M., Moldovan G.-L. (2022). Mechanisms of PARP1 Inhibitor Resistance and Their Implications for Cancer Treatment. NAR Cancer.

[13] Panzarino N.J., Krais J.J., Cong K., Peng M., Mosqueda M., Nayak S.U., Bond S.M., Calvo J.A., Doshi M.B., Bere M. (2021). Replication Gaps Underlie BRCA Deficiency and Therapy Response. Cancer Res..

[14] Buckley N.E., Hosey A.M., Gorski J.J., Purcell J.W., Mulligan J.M., Harkin D.P., Mullan P.B. (2007). BRCA1 Regulates IFN-γ Signaling through a Mechanism Involving the Type I IFNs. Mol. Cancer Res..

[15] Ouchi T., Lee S.W., Ouchi M., Aaronson S.A., Horvath C.M. (2000). Collaboration of Signal Transducer and Activator of Transcription 1 (STAT1) and BRCA1 in Differential Regulation of IFN-Gamma Target Genes. Proc. Natl. Acad. Sci. USA.

[16] Coquet J.M., Chakravarti S., Kyparissoudis K., McNab F.W., Pitt L.A., McKenzie B.S., Berzins S.P., Smyth M.J., Godfrey D.I. (2008). Diverse Cytokine Production by NKT Cell Subsets and Identification of an IL-17–Producing CD4^−^NK1.1^−^ NKT Cell Population. Proc. Natl. Acad. Sci. USA.

[17] Kreienkamp R., Graziano S., Coll-Bonfill N., Bedia-Diaz G., Cybulla E., Vindigni A., Dorsett D., Kubben N., Batista L.F.Z., Gonzalo S. (2018). A Cell-Intrinsic Interferon-like Response Links Replication Stress to Cellular Aging Caused by Progerin. Cell Rep..

[18] Surrallés J., De La Fuente M., García A., González M.A., López F., Tarsounas M., Cantó C., Gómez A., Tominari T., López J.A. (2020). The MRE11-RAD50-NBS1 Complex Is Required for the Resolution of Single-Strand DNA Gaps Generated by BRCA1-Deficient Cells. J. Biol. Chem..

[19] Liu Z., Du W., Liu Y., Chen H., Liang J., Jiang J., Hu Y., Wu L., Xia Z., Shi J. (2021). MRE11 Degradation of Single-Strand DNA Facilitates cGAS Activation, Independent of Its Nuclease Activity. Cell Rep..

[20] Cho M.-G., Kumar R.J., Lin C.-C., Boyer J.A., Shahir J.A., Fagan-Solis K., Simpson D.A., Fan C., Foster C.E., Goddard A.M. (2024). MRE11 Liberates cGAS from Nucleosome Sequestration during Tumorigenesis. Nature.

[21] González-Navajas J.M., Lee J., David M., Raz E. (2012). Immunomodulatory Functions of Type I Interferons. Nat. Rev. Immunol..

[22] Tiwari N., Kuppusamy P., Narayanan R., Pradhan S., Ray S., Ghosh S., Das S., Ghosh A., Liu X., Zhuang Y. (2020). BRCA1 Localizes at Promoters of Type I Interferon Signaling Molecules and Regulates Their Up-Regulation. Cell Rep..

[23] Gronauer R., Madersbacher L., Monfort-Lanzas P., Floriani G., Sprung S., Zeimet A.G., Marth C., Fiegl H., Hackl H. (2024). Integrated Immunogenomic Analyses of High-Grade Serous Ovarian Cancer Reveal Vulnerability to Combination Immunotherapy. medRxiv.

[24] Zimmermann M., Murina O., Reijns M.A.M., Agathanggelou A., Challis R., Tarnauskaitė Ž., Muir M., Fluteau A., Aregger M., McEwan A. (2018). CRISPR Screens Identify Genomic Ribonucleotides as a Source of PARP-Trapping Lesions. Nature.

[25] Kwon J., Bakhoum S.F. (2020). The Cytosolic DNA-Sensing cGAS-STING Pathway in Cancer. Cancer Discov..

[26] Sasaki N., Homme M., Kitajima S. (2023). Targeting the Loss of cGAS/STING Signaling in Cancer. Cancer Sci..

[27] Loughner C.L., Bruford E.A., McAndrews M.S., Delp E.E., Swamynathan S., Swamynathan S.K. (2016). Organization, Evolution and Functions of the Human and Mouse Ly6/uPAR Family Genes. Hum. Genom..

[28] Luo L., McGarvey P., Madhavan S., Kumar R., Gusev Y., Upadhyay G. (2016). Distinct Lymphocyte Antigens 6 (Ly6) Family Members Ly6D, Ly6E, Ly6K and Ly6H Drive Tumorigenesis and Clinical Outcome. Oncotarget.

[29] AlHossiny M., Luo L., Frazier W.R., Steiner N., Gusev Y., Kallakury B., Glasgow E., Creswell K., Madhavan S., Kumar R. (2016). Ly6E/K Signaling to TGFβ Promotes Breast Cancer Progression, Immune Escape, and Drug Resistance. Cancer Res..

[30] Ablasser A., Chen Z.J. (2019). cGAS in Action: Expanding Roles in Immunity and Inflammation. Science.

[31] Unterholzner L., Dunphy G. (2019). cGAS-Independent STING Activation in Response to DNA Damage. Mol. Cell. Oncol..

[32] Buis J., Wu Y., Deng Y., Leddon J., Westfield G., Eckersdorff M., Sekiguchi J.M., Chang S., Ferguson D.O. (2008). Mre11 Nuclease Activity Has Essential Roles in DNA Repair and Genomic Stability Distinct from ATM Activation. Cell.

[33] Lemaçon D., Jackson J., Quinet A., Brickner J.R., Li S., Yazinski S., You Z., Ira G., Zou L., Mosammaparast N. (2017). MRE11 and EXO1 Nucleases Degrade Reversed Forks and Elicit MUS81-Dependent Fork Rescue in BRCA2-Deficient Cells. Nat. Commun..

[34] Quinet A., Tirman S., Jackson J., Šviković S., Lemaçon D., Carvajal-Maldonado D., González-Acosta D., Vessoni A.T., Cybulla E., Wood M. (2020). PRIMPOL-Mediated Adaptive Response Suppresses Replication Fork Reversal in BRCA-Deficient Cells. Mol. Cell.

[35] Ding L., Wang Q., Martincuks A., Kearns M.J., Jiang T., Lin Z., Cheng X., Qian C., Xie S., Kim H.-J. (2023). STING Agonism Overcomes STAT3-Mediated Immunosuppression and Adaptive Resistance to PARP Inhibition in Ovarian Cancer. J. Immunother. Cancer.

[36] Jiang Y., Li S., Wang Y., Li Y., Wang W., Liu Y., Li Y., Ma J., Zhang L., Liu X. (2021). IFI16 and DNA Repair Proteins ATM and PARP1 Activate STING in a Non-Canonical Pathway. Nat. Commun..

[37] Huang Y., Zhao Y., Jiang J., Zhang X., Zhang H., Liu S., Zhang S., Zhang J., Li C., Lin X. (2023). STING Agonism Reprograms Myeloid Cells in the Tumor Microenvironment of PARPi-Resistant Ovarian Tumors and Overcomes Resistance. Cancer Immunol. Res..

[38] Yu D., Huang C.-J., Tucker H.O. (2024). Established and Evolving Roles of the Multifunctional Non-POU Domain-Containing Octamer-Binding Protein (NonO) and Splicing Factor Proline- and Glutamine-Rich (SFPQ). J. Dev. Biol..

[39] Petti E., Buemi V., Zappone A., Schillaci O., Broccia P.V., Dinami R., Matteoni S., Benetti R., Schoeftner S. (2019). SFPQ and NONO Suppress RNA:DNA-Hybrid-Related Telomere Instability. Nat. Commun..

[40] Ayers M., Lunceford J., Nebozhyn M., Murphy E., Loboda A., Kaufman D.R., Albright A., Cheng J.D., Kang S.P., Shankaran V. (2017). IFN-γ–Related mRNA Profile Predicts Clinical Response to PD-1 Blockade. J. Clin. Investig..

[41] Speiser D.E., Chijioke O., Schaeuble K., Münz C. (2023). CD4+ T Cells in Cancer. Nat. Cancer.

[42] Kawakami Y., Kumagai K., Kobayashi T., Saito T. (2019). CD4+ T Cells: Key Players in the Regulation of Immune Responses. Front. Immunol..

[43] Snyder A., Makarov V., Merghoub T., Yuan J., Zaretsky J.M., Desrichard A., Walsh L.A., Postow M.A., Wong P., Ho T.S. (2014). Genetic Basis for Neoantigen Burden as a Predictor of Clinical Benefit to Immune Checkpoint Blockade. Science.

[44] Kim C., Wang X.-D., Yu Y. (2020). PARP1 Inhibitors Trigger Innate Immunity via PARP1 Trapping-Induced DNA Damage Response. eLife.

[45] Benguigui M., Cooper T.J., Kalkar P., Schif-Zuck S., Halaban R., Bacchiocchi A., Kamer I., Deo A., Manobla B., Menachem R. (2024). Interferon-Stimulated Neutrophils as a Predictor of Immunotherapy Response. Cancer Cell.

[46] Wang Z., Sun K., Xiao Y., Feng B., Mikule K., Ma X., Feng N., Vellano C.P., Federico L., Marszalek J.R. (2019). Niraparib Activates Interferon Signaling and Potentiates Anti-PD-1 Antibody Efficacy in Tumor Models. Sci. Rep..

[47] Zhang L., Zhao Y., Liu J., Song W., Wang Q., Li Y., Zhang X., Xu L. (2016). CNV and Elevated Expression of PUF60 Are Associated with Cancer Progression and Poor Prognosis. Oncotarget.

[48] Beroukhim R., Mermel C.H., Porter D., Wei G., Raychaudhuri S., Donovan J., Barretina J., Boehm J.S., Dobson J., Urashima M. (2010). The Landscape of Somatic Copy-Number Alteration across Human Cancers. Nature.

[49] Matejcic M., Saunders E.J., Dadaev T., Brook M.N., Wang K., Sheng X., Al Olama A.A., Schumacher F.R., Ingles S.A., Govindasami K. (2018). Germline variation at 8q24 and prostate cancer risk in men of European ancestry. Nat. Commun..

[50] Zhou Z., Lu M., Wang Z., Yu L., Zhang L., Yan Y., Chen L., Zhang C., Chen L. (2018). LY6E Overexpression Promotes Cancer Cell Growth and Metastasis in Breast Cancer, Gastric Cancer, and Lung Cancer. Oncogene.

[51] Fultang L., Booth S., Yogev O., Danilo M., Singh S., Jin W., Xie C., Edwards N.J., Tarrats N., Jimenez L. (2020). LY6E Impacts T Cell Functions and Modulates Immune Checkpoint Signaling in Cancer. Nat. Commun..

[52] Zhou Q., Lin H., Wang S., Wang S., Yang H., Zhang H., Nie C., Yang C., Zhu M., Wang D. (2018). LY6E and IFI27 Are Upregulated in Response to Immune Activation via the cGAS–STING Pathway and Promote Interferon Production. Front. Immunol..

[53] Jiang D., Guo S., Wang X., Chen M., Zhang Y., Cao C., Zhang Q., Chen Y. (2020). IFI27 Is Localized to the Mitochondrial Membrane and Involved in Regulating Mitochondrial Integrity and Apoptosis. Cell Death Dis..

[54] Kuo C.Y., Chen J.H., Liu T.C., Huang Y.T., Chou Y.C., Wu Y.C., Chen T.W., Chang H.C., Chang S.Y. (2021). Altered Mitochondrial Dynamics and Energy Metabolism in PARPi-Resistant Ovarian Cancer Cells as Adaptive Mechanisms for Survival. Cancer Lett..

[55] Li T., Zhao J., Wang C., Wu Z., Liu X., Zhang L., Zhang H., Yang X. (2020). Overexpression of IFI27 Contributes to Drug Resistance and Apoptosis Resistance in Cancer Cells, Supporting Survival Despite Chemotherapy. Cancer Sci..

[56] Cheng H., Zhang H., Wu J., Wang J., Liu X., Liu C., Zhang X., Zhao Y., Zheng Y., Liu C. (2021). IFI27 Interacts with RAD21 to Regulate Nuclear DNA Damage Response and DNA Repair Mechanisms. Cell Rep..

[57] Hauf S., Waizenegger I.C., Peters J.M. (2001). Cohesin Cleavage by Separase Required for Anaphase and Cytokinesis in Human Cells. Science.

[58] Yan M., Xu H., Waddell N., Shield-Artin K., Haviv I., McKay M.J., Fox S.B., kConFab authors (2012). Enhanced RAD21 Cohesin Expression Confers Poor Prognosis in BRCA2 and BRCAX, but Not BRCA1 Familial Breast Cancers. Breast Cancer Res. BCR.

[59] Zhang C., Ni X., Tao C., Zhou Z., Wang F., Gu F., Cui X., Jiang S., Li Q., Lu H. (2024). Targeting PUF60 Prevents Tumor Progression by Retarding mRNA Decay of Oxidative Phosphorylation in Ovarian Cancer. Cell. Oncol..

[60] Sun D., Lei W., Hou X., Li H., Ni W. (2019). PUF60 Accelerates the Progression of Breast Cancer through Downregulation of PTEN Expression. Cancer Manag. Res..

[61] Klotz D.M., Schwarz F.M., Dubrovska A., Schuster K., Theis M., Krüger A., Kutz O., Link T., Wimberger P., Drukewitz S. (2023). Establishment and Molecular Characterization of an In Vitro Model for PARPi-Resistant Ovarian Cancer. Cancers.

[62] Hurley R.M., McGehee C.D., Nesic K., Correia C., Weiskittel T.M., Kelly R.L., Venkatachalam A., Hou X., Pathoulas N.M., Meng X.W. (2021). Characterization of a *RAD51C* -Silenced High-Grade Serous Ovarian Cancer Model during Development of PARP Inhibitor Resistance. NAR Cancer.

[63] Krämer A., Green J., Pollard J., Tugendreich S. (2014). Causal Analysis Approaches in Ingenuity Pathway Analysis. Bioinformatics.

[64] Tang Z., Kang B., Li C., Chen T., Zhang Z. (2019). GEPIA2: An Enhanced Web Server for Large-Scale Expression Profiling and Interactive Analysis. Nucleic Acids Res..

[65] Szklarczyk D., Kirsch R., Koutrouli M., Nastou K., Mehryary F., Hachilif R., Gable A.L., Fang T., Doncheva N.T., Pyysalo S. (2023). The STRING Database in 2023: Protein–Protein Association Networks and Functional Enrichment Analyses for Any Sequenced Genome of Interest. Nucleic Acids Res..

[66] Chen B., Khodadoust M.S., Liu C.L., Newman A.M., Alizadeh A.A. (2018). Profiling Tumor Infiltrating Immune Cells with CIBERSORT. Methods Mol. Biol. Clifton NJ.

