# Supplementary material for: Exploring Molecular Drivers of PARPi Resistance in BRCA1-Deficient Ovarian Cancer: The Role of LY6E and Immunomodulation
Source: Int J Mol Sci. 2024 Sep 27;25(19):10427. doi: 10.3390/ijms251910427 (PMC11477368; doi:10.3390/ijms251910427)
Supplement: Supplementary file 1 [file ijms-25-10427-s001.zip › ijms-3161823-supplementary.pdf]

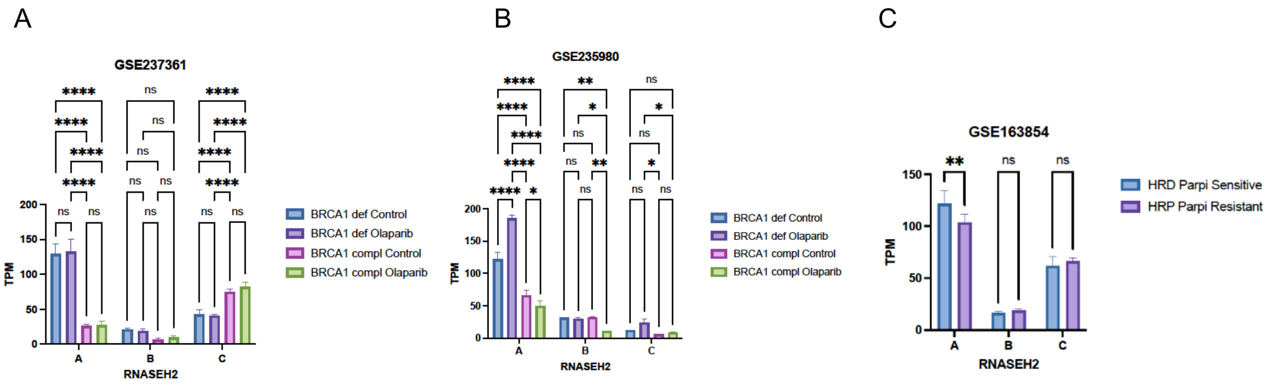

Supplementary Figure S1. Gene expression of the genes RNASEH2A, RNASEH2B, RNASEH2C in the three datasets according to the legend in each figure. **(A)** Gene expression from the dataset GSE237361, 96h after Olaparib treatment. **(B)** Gene expression from the dataset GSE235980, 11 months after Olaparib treatment. **(C)** Gene expression from the dataset GSE163854, PDX with 21 days after Olaparib resistance, where the cells sensitive are HR deficient (HRD) and the cells resistant are HR proficient (HRP). Two-way ANOVA multiple comparison test with adjusted *p*-value applied to the asterisks. Ns = $p > 0.05$; * $p \leq 0.05$; ** $p \leq 0.01$; *** $p \leq 0.001$ and **** $p \leq 0.0001$.

A

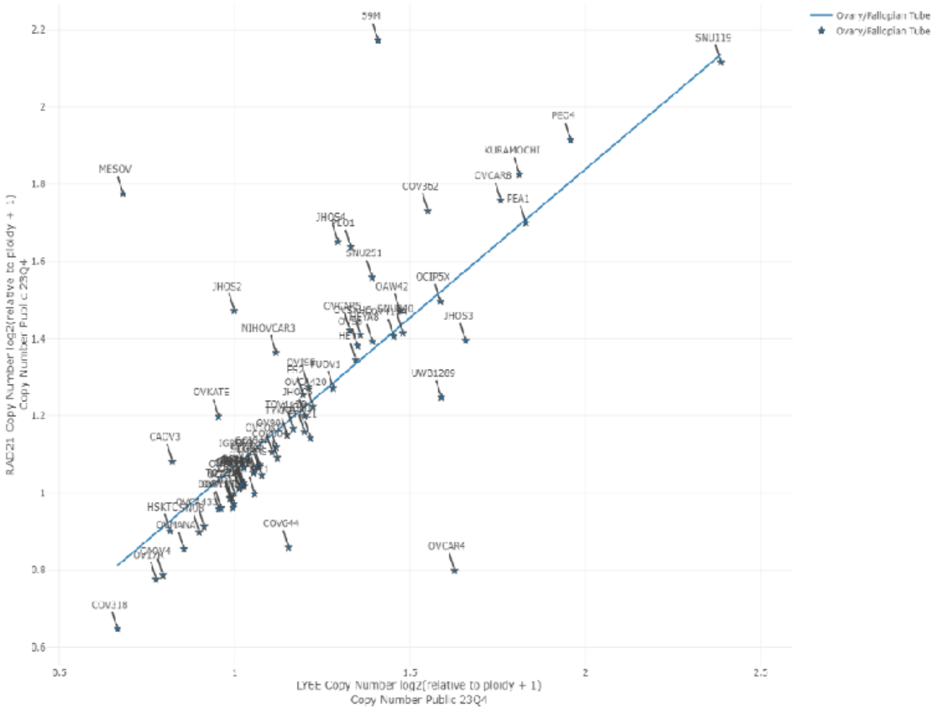

B

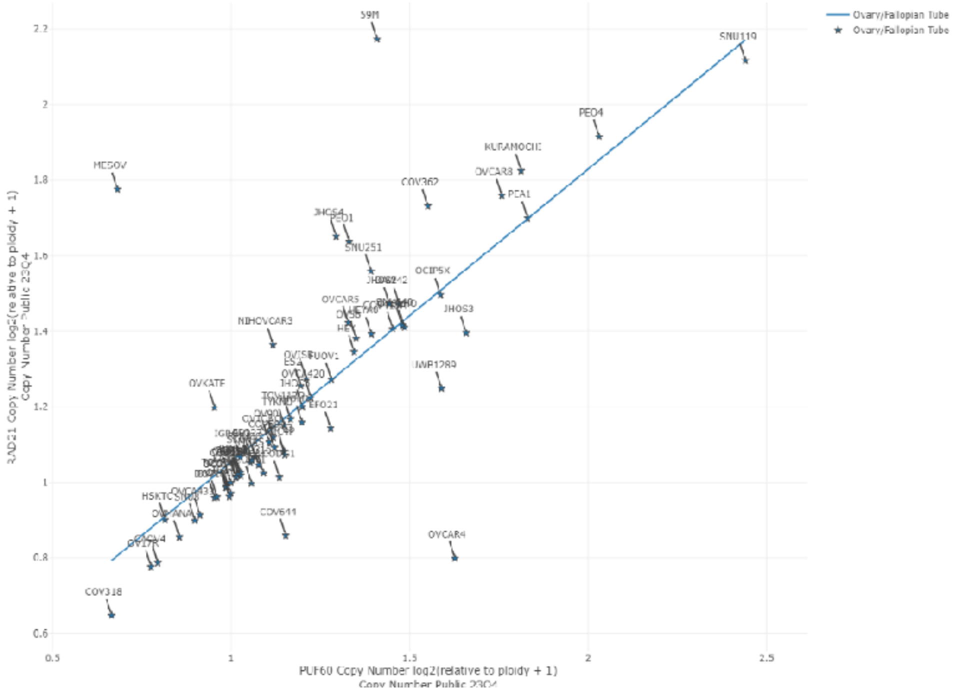

Supplementary Figure S2. Gene expression correlation in ovarian cancer cell lines in the depmap portal (depmap.org). The arrows show individual labels for all the ovarian cancer cells studied. The blue line show the regression line for their relationship. A. RAD21 and LY6E (Pearson 0.76, Spearman 0.79, p-value 1.56e-140) B. RAD21 and PUF60 (Pearson 0.74, Spearman 0.76, p-value 7.80e-14).
